# Limb-Sparing Surgery and Stifle Arthrodesis Using Patient-Specific 3D-Printed Guides and Endoprosthesis for Distal Femoral Chondrosarcoma in a Dog: A Case Report

**DOI:** 10.3390/ani15050673

**Published:** 2025-02-26

**Authors:** Enrico Panichi, Marco Tabbì, Gaetano Principato, Valentina Dal Magro, Fabio Valentini, Marco Currenti, Francesco Macrì

**Affiliations:** 1 Centro Traumatologico Ortopedico Veterinario, Via C. Festa 9, 16011 Arenzano, GE, Italy; enricopanichi76@gmail.com (E.P.); valentinadalmagro@gmail.com (V.D.M.); marcocurrenti.vet@gmail.com (M.C.); 2 Department of Veterinary Sciences, University of Messina, Polo Universitario dell’Annunziata, 98168 Messina, ME, Italy; gaetanoprincipato@gmail.com (G.P.); francesco.macri@unime.it (F.M.); 3 Servizio di Oncologia Veterinaria ONCOVET, Via Chisimaio, 32, 00199 Roma, RM, Italy; f.valentini@oncovet.it

**Keywords:** chondrosarcoma, limb sparing, virtual surgical planning, 3D printing, patient-specific guides, patient-specific endoprosthesis

## Abstract

Limb-sparing techniques for appendicular primary bone tumors are still associated with a high rate of complications. Three-dimensional (3D)-printed patient-specific instruments could reduce these complications. The aim of this study is to describe a limb-sparing surgery using 3D-printed patient-specific guides (PSGs) and an endoprosthesis (PSE) to treat femoral chondrosarcoma in a dog. An eight-year-old female Golden Retriever presented with persistent lameness of the right hind limb, reluctance to move and difficulty in maintaining a standing position. Palpation of the right femur revealed an approximately 4 cm painful lesion. Cytological analysis of the needle aspiration supported the clinical and radiological suggestion of a cartilaginous bone neoplasm. Computed tomography (CT) scans suggested the presence of an aggressive lesion on the right distal femur. CT scans of the femur and tibia were then reconstructed using a bone tissue algorithm and processed with computer-aided design (CAD) software, which allowed for performing virtual surgical planning (VSP) and the fabrication of both the PSG and the PSE. Anti-inflammatory drugs and monoclonal antibodies were used for pain management while waiting for surgery. Adjuvant chemotherapy was also administered. An ostectomy of the distal third of the femur to completely remove the tumor was performed with the designed PSG, while the bone defect was filled with the designed PSE. Histopathological examination of the osteotomized bone segment confirmed a grade 2 central chondrosarcoma. There was no excessive tumor growth during the 28 days between the CT scans and surgery. Both PSG and PSE fitted perfectly to the bone surfaces. PSG eliminated the need for intraoperative imaging and ensured a faster and more accurate osteotomy. PSE optimized load sharing and eliminated the complications of the commercial endoprosthesis, such as incongruity and the need for manual intraoperative adjustment. Overall, the use of VSP, 3D-printed PSG and PSE significantly reduced surgical time, risk of infection and intra- and postoperative complications.

## Simple Summary

This study describes a limb-sparing surgical technique using virtual surgical planning and three-dimensional printed patient-specific guides and endoprosthesis to treat a femoral chondrosarcoma in a dog. The computer-aided design software reported in this study allowed tumor assessment for implant design, virtual surgical planning and simulation of the ostectomy. Patient-specific osteotomy guides reduced intraoperative time, eliminated the need for intraoperative diagnostics and ensured a more accurate ostectomy. The patient-specific endoprosthesis reduced intraoperative time and surgical complications and allowed for shorter postoperative recovery time. The combination of virtual surgical planning with the use of three-dimensional-printed patient-specific guides and an endoprosthesis resulted in a favorable clinical outcome. Weight-bearing was observed in the patient already two days after surgery.

## 1. Introduction

Chondrosarcoma (CSA) is the second most common bone tumor in both dogs and humans, accounting for almost 10% of reported primary bone tumors in dogs [1,2,3]. The nasal cavities and axial and appendicular skeleton, as well as extraskeletal sites, are the reported primary sites [4]. There have been several reports of skeletal CSA, but only a small number of appendicular cases have been reported in the literature [2,4]. Appendicular CSA usually affects adults and older animals between 5.9 and 8.7 years of age and most commonly occurs in medium- and large-breed dogs weighing between 20 and 40 kg [1,2]. Boxers, German Shepherds and Golden Retrievers are the most predisposed breeds, with Golden Retrievers having an approximately 3.12 times higher risk of developing CSA than any other breed [5].

Total limb amputation is considered the standard of care in dogs for appendicular tumors but also for other distal limb pathologies. Although frequently performed in humans, partial limb amputation followed by socket prostheses is still emerging in veterinary medicine [1,2], with limited objective data available and many questions regarding the use of prostheses and the high complication rate observed in canine patients [6,7]. Partial or complete limb amputation has been the surgical treatment of choice for many years because it is relatively simple to perform and allows for the complete removal of the tumor with minimal risk of complications and infection. However, this approach can aggravate pre-existing orthopedic conditions or predispose to them, so not all patients are good candidates. In addition, some dog owners refuse amputation because they are concerned that it will affect their pet’s quality of life (QOL) [8].

Limb-sparing surgery is a valid alternative to amputation that can preserve QOL in patients who are expected to have significant difficulty walking after limb amputation due to orthopedic conditions, such as degenerative joint disease (DJD) in the unaffected limb, neurological disease or large body size [9,10]. Limb-sparing surgery is a salvage technique that involves removing the primary tumor of bone and applying internal or external fixation to the remaining bones, with or without segmental bone substitution. Several limb-sparing surgical techniques have been described in veterinary medicine for appendicular tumors. Traditional limb-sparing surgery involves tumor resection and reconstruction of the bony column with commercially available implants, while some of the alternative limb-sparing techniques include stereotactic radiosurgery (STS), pasteurized autografts, irradiated autografts, ulnar rollover transposition (URT), lateral manus translation (LMT) and bone transport osteogenesis. However, the described limb-sparing techniques are associated with various complications, such as infection (up to 80%), deterioration of bone quality with increased fragility and risk of pathological fractures, local recurrence (up to 60%) and implant rupture and failure (up to 60%), which, in most cases, require total limb amputation. Furthermore, limb-sparing procedures using conventional techniques and commercially available implants can have significant limitations in terms of both surgical precision and functional outcome [9,10,11,12].

Conventional bone oncology surgery is primarily based on two-dimensional planning and surgeon experience, which can lead to inaccurate resection margins and difficulties in anatomical reconstruction [13,14]. Computer-aided design (CAD) software, virtual surgical planning (VSP) and three-dimensional (3D)-printing technology have revolutionized many areas of human surgery, enabling the creation of patient-specific instruments (PSIs), such as patient-specific cutting guides (PSGs) and endoprosthesis (PSE) reconstructed directly from the patient’s CT scan [15,16,17,18]. Precise 3D planning of tumor resection for oncological clearance and design of PSIs for the perfect reconstruction of bony defects are the main advantages of 3D printing in limb-sparing procedures. The reduction of surgery time and related complications, such as infection rate; the reduction of associated costs; and the improvement of patients’ postoperative prognosis, recovery and implant-related failures are also important indirect advantages. Despite promising results in human medicine, the application of these innovative technologies in veterinary surgical oncology remains limited and poorly documented, with only a few examples reported in the literature [19,20,21,22].

To the author’s knowledge, this is the first case report describing a limb-sparing surgery using 3D-printed patient-specific guides (PSGs) and an endoprosthesis (PSE) for the treatment of femoral CSA in a dog.

## 2. Case Description

### 2.1. Patient History and Clinical Findings

An eight-year-old female Golden Retriever was referred to the Centro Traumatologico Ortopedico Veterinario (C.T.O. VET., Arenzano, Italy) for specialized evaluation. The owners reported persistent lameness of the right hind limb, reluctance to move and difficulty in maintaining a standing position. The clinical history revealed a condition characterized by progressive lameness with pain in the femur and stifle, present at rest, even at night, and aggravated by moderate physical activity or owner manipulation. A complete orthopedic examination was performed. Lameness was scored by observing the patient at rest and in motion, including gait and trot scores. During observation, raising the ischial tuberosity on weight-bearing on the right hind limb (hip strike) was noted. Lameness was graded as grade III according to the previously described lameness scoring system [23]. Palpation of the right femur revealed a subcutaneous round lesion approximately 4 cm in diameter, hard, well demarcated from the surrounding tissues, not pedunculated, not very mobile and well adherent to the underlying tissues. The skin surface was intact without ulceration or abrasions, but the area was swollen, warm and painful to palpation. The range of motion (ROM) of the affected joint was then assessed to determine any flexion–extension limitations, with marked pain and flexion ROM reduction. In addition, muscle trophism was compared with the contralateral limb, with mild disuse muscle hypotrophy observed in the affected limb. A neurological examination was also performed, focusing on postural and proprioceptive responses. The examination did not reveal any significant deficits, thus excluding any neurological impairment. Finally, a general clinical examination and blood sampling for hematologic and biochemical analysis were performed to exclude the presence of any concomitant systemic disease. A complete blood count (CBC) and biochemical evaluation (azotemia, urea, transaminases and total protein) were performed. The results revealed no concomitant pathology.

### 2.2. Radiographic Study

**Figure 1:**
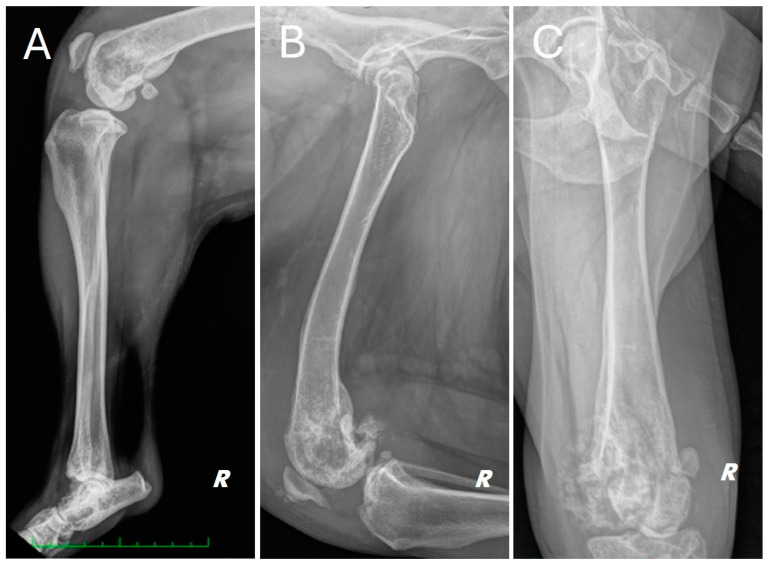
Different radiographic projections of the right hind limb. (**A**) mediolateral projection of the tibia, (**B**) mediolateral projection of the femur, (**C**) cranio-caudal projection of the femur. Structural and radiopacity alteration near the distal femoral end and the knee joint is repeated in all projections performed.

Radiographs of the right hind limb were taken in mediolateral and craniocaudal projections under sedation and analgesia (Figure 1).

The radiograph showed evidence of osteoarthritis of the right hip joint with bone remodeling of the femoral head and neck. Significant structural changes were observed in the distal metaphysis of the right femur, with a marked “moth-eaten” pattern of bone lysis, pronounced and irregular periosteal reaction and extensive alteration of the normal pattern of the medullary canal in a disto-proximal direction up to the level of the middle third of the diaphysis. A complete, comminuted, slightly dislocated pathological joint fracture of the medial femoral condyle, osteoarthritis of the distal pole of the patella and marked joint and periarticular soft tissue swelling of the femorotibial–patellar joint were also found. Finally, there was moderate sclerosis and mild osteophytosis of the tibiotarsal joint.

### 2.3. Cytologic Examination

To obtain a definitive diagnosis of the nature of the lesion, an echo-guided needle aspiration for cytological analysis was performed after a trichotomy and surgical scrubs of the area of interest. The material obtained was then applied on glass slides, smeared and sent to the laboratory for staining and reading. The laboratory report described the presence of spindle cells with moderately eosinophilic cytoplasm, indistinct borders, large central round or oval nuclei with fine chromatin and moderate-sized, predominantly single nucleoli. Moderate anisocariasis and anisocytosis were also observed, as well as occasional binucleated cells. The degree of anaplasia that was found supported the clinical and radiographic suggestion of a bone neoplasm compatible with a sarcoma, probably of cartilaginous origin.

### 2.4. Advanced Diagnostics: Total Body CT Scan

**Figure 2:**
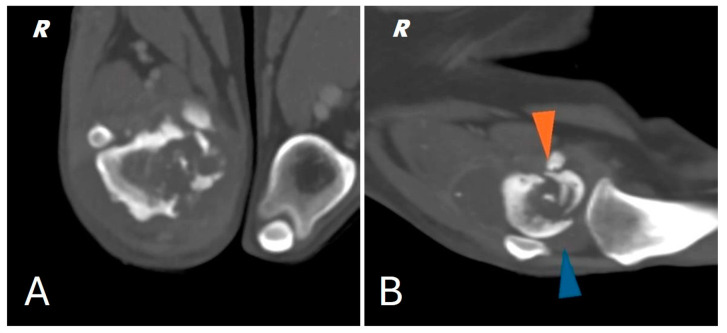
(**A**) CT scan of the right distal femur. (**B**) CT scan of the knee joint; pathologic fracture of the medial femoral condyle (orange arrowhead) joint effusion (blue arrowhead).

A total body computed tomography (CT) study using a 16-slice helical scanner (Somatom Emotion 16; Siemens, Erlangen, Germany) was performed to determine the extent of the neoplasm and its borders, the degree of cortical proliferation and soft tissue infiltration and the presence of any pulmonary metastases that were not visible on conventional radiography. The patient was sedated with a combination of intravenous dexmedetomidine (Dexdomitor, Vétoquinol Italia S.r.l., Bertinoro, Italy; 0.005 mg/kg) and butorphanol (Dolorex, MSD Animal Health S.r.l, Milan, Italy; 0.3 mg/kg) and then positioned in sternal recumbency with both thoracic limbs extended cranially and both pelvic limbs extended caudally. The CT acquisition parameters were 120 kVp and 150 mAs. A helical total body scan was reconstructed in a pre- and post-contrast 1 mm standard algorithm, and a 0.6 mm scan from the ilium to the toes was reconstructed using the bone algorithm. Contrast was administered IV at a dose of 770 mg iodine/kg of non-ionic contrast (Omnipaque, 350 mg iodine/mL, GE Healthcare Inc., Marlborough, MA, USA). After the acquisition of the planning CT study, the surgeon evaluated the tumor with a radiologist. The examination confirmed the presence of an aggressive lesion localized to the right distal femur, characterized by marked bone lysis, pronounced periosteal reaction and extensive increase in intramedullary density extending into the middle third of the diaphysis of the femur (Figure 2).

In addition, CT showed a complete comminuted and slightly dislocated fracture of the medial femoral condyle ipsilateral to the injury, with marked swelling of the joint and periarticular soft tissues extending to the course of the long digital extensor, hypomyotrophy of the right limb with mild lymphadenomegaly of the medial iliac and sacral lymph nodes on the same side. Multifocal osteoarthrosis involving the elbow, tarsi and coxofemoral joints with mild bony remodeling, smooth margins and mild joint tumefaction were also noted. The CT scan showed no evidence of pulmonary metastases.

### 2.5. Design and 3D Printing of Patient-Specific Devices

**Figure 3:**
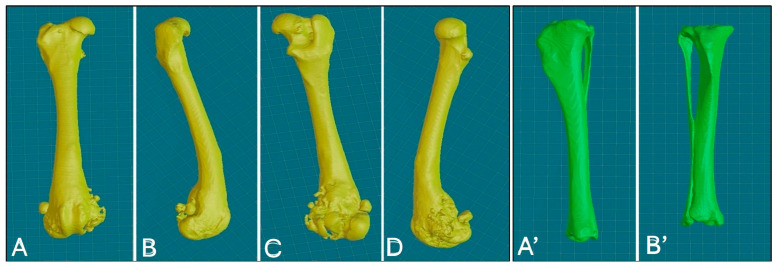
Three-dimensional reconstruction of the right femur, various views: cranial (**A**), lateral (**B**), caudal (**C**), medial (**D**); 3D reconstruction of the right tibia, various views: caudal (**A’**), cranial (**B’**).

High-resolution CT scans of the femur and tibia were used for the 3D printing of PSGs and PSE. DICOM (digital imaging and communications in medicine) images were reconstructed with a slice thickness of 0.6 mm using a bone tissue algorithm and processed with CAD software (BonaPlanner v1.1.0, ©Bonabyte, Moscow, Russia). A bioengineer supervised the segmentation process, which allowed for the accurate 3D reconstruction of the bones; manipulation of the femur and tibia in the axial, sagittal and frontal planes; and measurement of the margins for bone segment removal (Figure 3).

**Figure 4:**
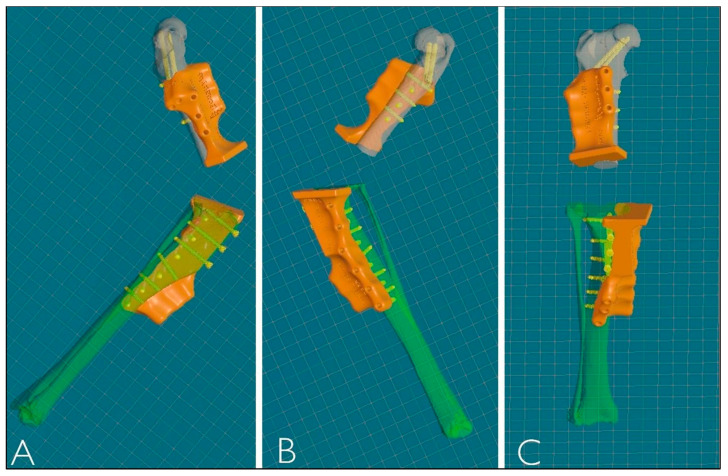
Three-dimensional reconstruction of PSGs. (**A**) Lateral view. (**B**) Medial view. (**C**) Frontal view.

After reconstruction of the femur and tibia, VSP was performed to simulate the surgery using the same CAD software. This allowed the femur and tibia to be visualized and manipulated in all three planes during all phases of the virtual ostectomy to optimize the result. Stereolithographic 3D printing (SLA) was used to produce the osteotomy guides in Nextdent Dental SG material, designed according to the surgeon’s blade parameters and the diameters of the selected screws. The guides, modeled with 3D contours corresponding to the complementary shape of the bone, provided precision in positioning, cutting angles and patient-specific implant fit, ensuring the stability of the osteotomy site (Figure 4).

The femoral osteotomy line was drawn to obtain a transverse osteotomy with respect to the anatomical femoral axis in the sagittal and frontal planes, 5 cm from the proximal end of the lesion highlighted on the CT scan. The first tibial osteotomy line was traced to obtain better visualization of the knee joint and to remove the intra-articular soft tissues (e.g., meniscus and cruciate ligaments). The second tibial osteotomy line was traced to remove a thin slice of bone with a cut parallel to the tibial plateau to allow contact of the cancellous bone with the porous material present on the endoprosthesis to promote osteointegration.

**Figure 5:**
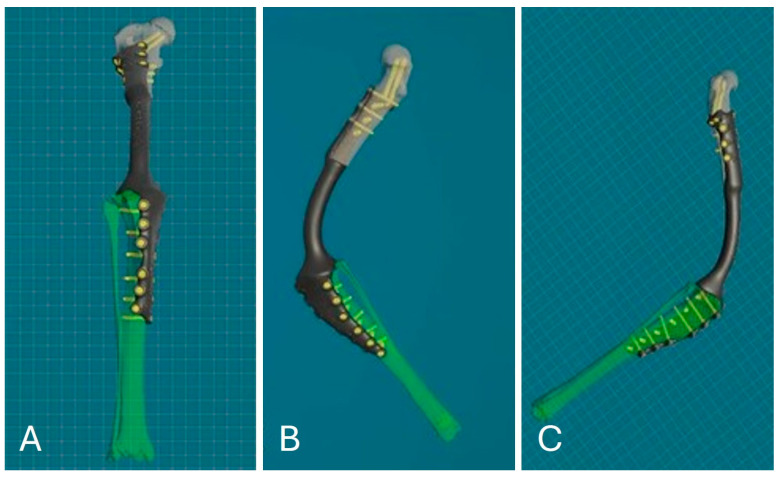
PSE 3D reconstruction: frontal view (**A**); medial view (**B**); lateral view (**C**).

The titanium alloy prosthesis to replace the ostectomized bone portion was designed using powder bed additive manufacturing (PB-AM) techniques with electron beam melting (EBM), using a Ti-6Al-4V alloy in accordance with ASTM and US FDA standards for surgical implants [24,25]. The surgeon selected the location and diameter of the screws, with a minimum of 8 screws proximally and 12 distally in a polyaxial configuration (three screws cranio-caudal, three screws latero-medial and two screws latero-medial and disto-proximal towards the femoral head; six screws cranio-caudal and six screws medio-lateral on the right tibia) to ensure implant stability as in a type 1b external skeletal fixation configuration. The desired angle of stifle flexion for arthrodesis was determined based on the position of the contralateral stifle in the standing position from a lateral view. The arthrodesis angle was 120°, defined by two lines—the anatomical axis of the femur and tibia in the sagittal plane. The cylindrical part of the prosthesis was designed with the same length and procurvatum as the normal femur to avoid any hipometry. An appendicular porous stem was also designed to fit into the medullary cavity of the distal ostectomized femur, ending under the last distal femoral prosthesis screw. Based on the analysis of the density and biomechanical properties of the bone heads, the bioengineer then adjusted the position of the screws to ensure reliable fixation. The shape, thickness, length and width of the prosthesis were defined from the CT scans based on the bone diameters, faithfully reproducing the removed bone segment and adapting to the patient’s size and planned screw configuration (Figure 5).

### 2.6. Limb-Sparing Surgery

While awaiting surgery, pain was managed with anti-inflammatory drugs—firocoxib every 24 h (Previcox, Boehringer Ingelheim, Milan, Italy; 5 mg/kg) and the monoclonal antibody bedinvetmab every 28 days (Librela, Zoetis, Modena, Italy; 1 mg/kg). Adjuvant chemotherapy was given with carboplatin at 400 mg/m2 body surface area, administered intravenously every 4 weeks. Blood tests were performed concurrently to monitor for potential myelosuppression, which did not occur.

The anesthetic protocol included premedication with acepromazine (Prequillan, Fatro SpA, Bologna, Italy; 0.02–0.04 mg/kg) and methadone (Semfortan, Eurovet Animal Health BV, Bladel, The Netherlands; 0. 2 mg/kg), induction with propofol (Propovet Multidose, Zoetis Srl, Rome, Italy; 4 mg/kg EV) and maintenance with sevoflurane (SevoFlo, Zoetis Belgium SA, Louvain-la-Neuve, Belgium). Intraoperative analgesia was achieved with fentanyl (Fentadon, Eurovet Animal Health BV, Bladel, The Netherlands; 2–4 µg/kg IV) at a constant infusion rate. Prophylactic antibiotic treatment with cefazolin (Cefazolin TEVA, Milan, Italy; 20 mg/kg) was administered intravenously 30 min before surgery and repeated every 90 min until the end of the surgical procedure. Monitoring included recording of heart and respiratory rate, end-expiratory CO_2_, blood O_2_ saturation and non-invasive blood pressure.

The patient underwent an extensive trichotomy of the entire right hind limb from the distal metatarsus to the dorsal midline and was positioned in dorsal recumbency with the possibility of lateral tilt. Surgical scrubbing was performed to prepare the surgical field. The surgical approach used is a combination of the three surgical approaches previously described [26]. In chronological order, the surgical approaches performed were the approach to the shaft of the femur, the approach to the distal femur and stifle joint by osteotomy of the tibial tuberosity and the approach to the shaft of the tibia.

The skin incision was made along the craniolateral edge of the femur from the greater trochanter to the distal pole of the knee joint. The skin incision was continued on the cranial side of the stifle to the medial side of the proximal tibia and extended distally to one-third of the length of the tibia. The subcutaneous fat and superficial fascia were incised beneath the skin incision. The skin margins were retracted, and the fascia lata was incised along the cranial border of the biceps femoris muscle. The femur was exposed by caudal retraction of the biceps femoris and cranial retraction of the vastus lateralis and vastus intermedius muscles. On the tibia, the subcutaneous tissue was incised to locate the pes anserinus muscles, which were incised on the tibia and continued distally on the tibial diaphysis and then separated from the underlying joint capsule and medial collateral ligament. The medial edge of the popliteus insertion was incised proximodistally, lifting and retracting the muscle origin.

**Figure 6:**
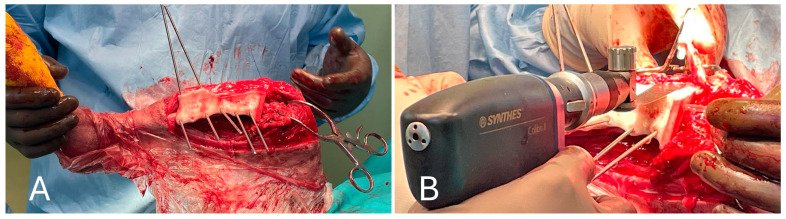
(**A**) Positioning of the cutting guide on the tibia and preparation of the tibial tuberosity osteotomy; (**B**) performing the tibial tuberosity osteotomy and stifle visualization after medial patellar tendon retraction.

Medial and lateral parapatellar arthrotomies were performed. They were first made with the scalpel on the medial side and then on the lateral side, starting at the distal pole of the patella and perpendicular to the patellar ligament. A stab incision was made at the proximal end to gain access to the articular cavity. Scissors were then inserted into the joint and advanced proximally, cutting the articular capsule and lateral parapatellar fibrocartilage. The same was performed on the lateral side of the patellar ligament. The proximal incision was directed slightly laterally to cut the vastus lateralis muscle parallel to the muscle fibers, leaving sufficient tissue on both sides of the patella for suturing. Therefore, the soft tissues adhering to the bone in the proximal, medial part of the tibia were removed (medial collateral ligament and articular capsules), the tibial cutting guide was placed with a 2.5 Stainmann pin and the tibial tuberosity was then osteotomized to allow for the proximal retraction of the entire quadriceps group. Distal retraction of the fat pad exposed the cruciate ligaments and menisci. After exposure of the knee joint, the cruciate ligaments, meniscus–femoral ligaments, long digital extensor and menisci were removed (Figure 6).

**Figure 7:**
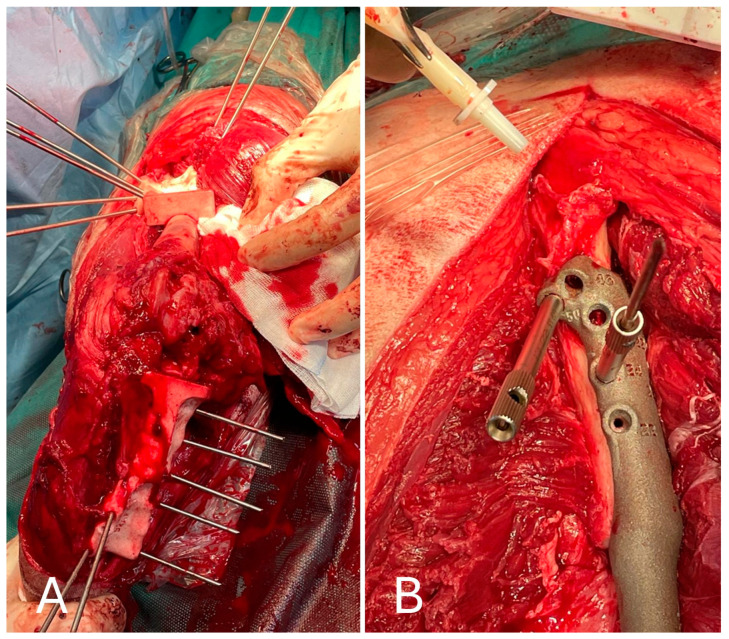
(**A**) Cranial view of the tibia after the tibial tuberosity osteotomy; (**B**) placement of the implant in the proximal femur.

After inspection of the neoplastic bone segment, the 3D-printed patient-specific femoral osteotomy guide was positioned and fixed to the bone with 2.5 mm Steinmann pins corresponding to the diameter of the guide holes. With the guide correctly positioned, the femoral osteotomy was performed parallel to the surface of the guide using an oscillating saw. Once the femoral osteotomy was complete, the guide was removed. Similarly, the osteotomy of the tibial plateau was performed following the tibial cutting guide as planned in the virtual surgical planning. Any residual irregularities of the bone surfaces were removed by careful trimming with an oscillating saw. The neoplastic portion of the femur was removed en bloc, and the femoral canal was reamed to prepare it to receive the prosthesis stem, allowing the titanium endoprosthesis to be placed. The prosthesis was fixed with 3.5 mm titanium locking screws inserted into the prepared holes using a drill and locking guide. The implant was placed in the bone defect and anchored to the cranio-lateral surface of the proximal third of the femur and the cranio-medial surface of the proximal tibia (Figure 7).

The tibial tuberosity was then fixed in its anatomical position with two 1.2 mm Kirschner wires, together with the insertion of the patellar ligament, to ensure that the position of the quadriceps muscle mechanism was not altered after osteosynthesis with the customized implant. After thorough irrigation and placement of an active drain, the soft tissues were sutured using standard surgical techniques.

### 2.7. Postoperative Care

A modified Robert-Jones-type bandage was applied to the operated limb and maintained for 48 h to limit postoperative swelling. The patient was admitted to the hospital for 24 h and started postoperative supportive care. During the first 24 h after surgery, the subject received antibiotic treatment with cefazolin (Cefazolin TEVA, Milan, Italy; 20 mg/kg, EV q12 h) and analgesic treatment with methadone (Semfortan Eurovet Animal Health B.V., Handelsweg, The Netherlands; 0.2 mg/kg, IM, q6 h). Both antibiotic and analgesic treatments were then replaced by oral administration of cefadroxil (Cefa-Cure Tabs cpr, MSD Animal Health S.r.l., Milan, Italy; 20 mg/kg, q24 h for 7 days) and tramadol (Altadol, Formevet S.r.l., Milan, Italy; 2–4 mg/kg, q8 h), respectively. Postoperative analgesia was adapted to the clinical course and needs of the patient. In addition, oral anti-inflammatory therapy with meloxicam (Metacam, Boehringer Ingelheim Animal Health Italy S.p.A., Noventana, Italy; 0.2 mg/kg on the first day, then 0.1 mg/kg, q24 h for 10 days) and gastroprotective therapy (omeprazole at 1 mg/kg and sucralfate at 50 mg/kg, both OS for 15 days) were administered.

### 2.8. Histopathological Examination

The laboratory report described an infiltrating neoplastic proliferation extending from the medullary to the cortical bone with resorption of bone tissue. The lesion consisted of chondroblastic cells with increased cellularity, variable cell density and the formation of multifocal aggregates. Neoplastic cells were organized in a carpet pattern within an abundant hyaline cartilage matrix. Resorption of medullary trabecular bone tissue was also noted. Mitotic counts were less than one mitosis per 10 microscopic fields at high magnification. The results of the histopathological examination confirmed the clinical, radiographic and cytological diagnosis of a cartilaginous neoplasm without neoplastic osteoid matrix in the analyzed sections, indicating an intermediate-grade central CSA consistent with histological grade 2.

### 2.9. Follow-Up

**Figure 8:**
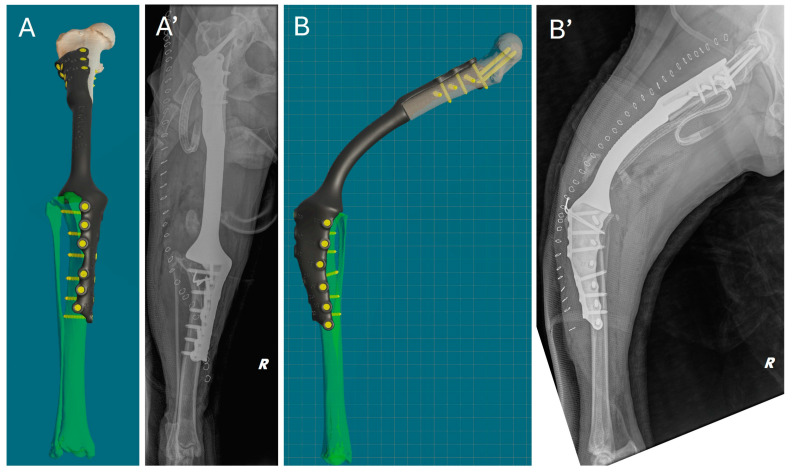
Comparison of virtual surgical planning and postoperative radiographs. (**A**,**A’**) Cranial view. (**B**,**B’**) Lateral view.

Immediately after surgery, a clinical assessment of joint stability and limb alignment was performed, and radiographs were taken in both standard orthogonal projections to assess the correct positioning of the implants and limb alignment. Correct implant positioning and limb alignment according to the preoperative plan were confirmed by the postoperative radiograph. (Figure 8).

Weight-bearing was observed in the patient already two days after surgery, with grade II (II/IV) of lameness at walk. At this time, the active drainage had been removed. Owners were given strict instructions to follow during the postoperative period, such as keeping the patient in a confined space and avoiding sudden exertion or jumping from any height. Walking was limited to short, leashed sessions of no more than ten minutes. At ten days after surgery, the skin suture staplers were removed, and the gait was clinically evaluated, with grade II (II/IV) lameness score at walk.

Clinical follow-up at 3, 6 and 12 months showed no signs of complications. At the 3-month postoperative evaluation, grade II of lameness persisted, accompanied by some circumduction of the limb at faster gaits (Video S1). However, no pain was elicited during manipulations. At the last clinical follow-up at 12 months, no infection, limb misalignment or other complications were observed, and no local tumor recurrence occurred. In addition to the clinical follow-ups, a final telephone follow-up was conducted at 18 months. The owner reported the dog’s behavior had improved, with a newfound enthusiasm for walks and a happier disposition, and that the patient was maintaining a normal lifestyle, except for the onset of fatigue after long walks. The absence of pain to palpation, visible swelling or lesions around the endoprosthesis was confirmed by clinical examination at another veterinary facility.

## 3. Discussion

The advancement of 3D-printing technology in veterinary medicine has revolutionized the approach to limb-sparing procedures. This study describes a limb-sparing technique using 3D-printed PSGs and PSE to treat femoral CSA in an eight-year-old canine patient.

Amputation has been the standard surgical approach to the treatment of primary appendicular bone tumors in both dogs and humans for many years. However, not all patients are good candidates due to several factors that can lead to significant morbidity and impact on QOL [8,27]. After amputation, the remaining limbs are forced to bear a greater proportion of the load and strain to support the animal’s total weight. The amputation of a forelimb is compensated for by a shift in the center of gravity and a redistribution of weight to the contralateral limb and the hind limbs, and vice versa. This leads to significant changes in gait, as the remaining limbs have to carry a greater load than is physiological. The distribution of body weight between the fore and hind limbs differs between breeds. Under physiological conditions, non-athletic breeds carry approximately 60% of their body weight on the forelimbs and 40% on the hindlimbs [27]. These differences in compensatory load redistribution after forelimb and hindlimb amputation result in a greater load on the remaining forelimb than on the hindlimb after amputation [8]. Ground reaction forces (GRFs) measured using force plate analysis have shown that dogs with forelimb amputations have reduced braking ability, coordination problems and greater difficulty maintaining balance, whereas dogs with hindlimb amputations have more difficulty gaining speed but are usually able to maintain balance and coordination [8]. These changes in gait could significantly increase the incidence of orthopedic conditions such as osteoarthritis (OA) in the remaining limbs, particularly in large or giant breeds [8]. Gait changes due to amputation warrant a thorough assessment of the remaining limbs, especially if patients have pre-existing orthopedic or neurological conditions. Orthopedic disease is not only a possible consequence of amputation but also a pre-existing condition that contraindicates amputation, as in our case of multifocal mild osteoarthritis of both elbows and hips. Large and giant breeds, which are commonly affected by CSA, are also commonly affected by degenerative joint disease (DJD) [1,2,5]. In our study, the radiographs showed evidence of DJD of the right hip joint and the distal pole of the patella. Bone remodeling of the femoral head and neck; marked joint and periarticular soft tissue swelling of the femorotibial–patellar joint; and moderate sclerosis and mild osteophytosis of the tibiotarsal joint were also noted. Given the patient’s pre-existing orthopedic condition, limb-sparing surgery was a more appropriate choice than amputation. Owner concerns about amputation surgery are another important factor to consider, as many owners oppose amputation as a treatment for many reasons [28]. Owners of amputated dogs reported a slight decrease in recreational activities (20%) and endurance during exercise (38%). Behavioral changes such as anxiety, fear and aggression towards other dogs, decreased dominance and lack of interest in other dogs were also reported (9% to 32%) [29]. Although it is impossible to determine the reason for these behavioral changes, it has been suggested that the change in the functional status of a three-legged dog may lead to difficulty in defending itself and a lower position in the hierarchy. In addition, owners are often concerned about how a three-legged dog will adapt to locomotion. This concern may be exacerbated by the effects of similar surgeries on humans and any doubts expressed by the veterinarian about the patient’s ability to adapt to the loss of a limb [27]. In our case, the owner of the animal was duly informed of the treatment options available, including chemotherapy alone and limb amputation. However, from the very beginning, the owner expressed serious concerns about his animal’s ability to maintain the QOL it had prior to the onset of clinical signs. Therefore, in consultation with the attending surgeon and due to DJD in the hips and elbows, the limb-sparing technique was chosen.

Limb-sparing surgery allows limb function to be preserved in cases of bone tumors or severe fractures and can improve long-term functionality [10,11,12,28]. Limb-sparing surgery involves ostectomy of the affected bone segment, followed by placement of an implant to bridge the remaining bone structures. In the conventional limb-sparing technique, this bridge can be made with bone grafts, such as cortical bone allografts or autografts, or with metal plate endoprosthesis. The main limitations of bone grafts are intraoperative irradiation or pasteurization, resulting in prolonged surgical time for autografts or costly and time-consuming canine bone banks for allografts [17]. The use of all-metal plate endoprosthesis may be an alternative, but there are only a few types and a very limited number of sizes commercially available for limb-sparing surgery in dogs. As a result, these commercially available implants require intraoperative contouring to fit the remaining bone structure with extremely limited degrees of freedom. These tasks prolong surgery and increase the number of intra/extra-corporeal manipulations, potentially increasing the risk of infection but also the risk of implant/bone failure and screw loosening/fracture (36–40%) [11,12]. Providing a patient-specific approach and implant could reduce intraoperative contouring and surgical time, reduce the risk of infection and maximize fit in the implantation site, increasing resistance to implant fracture or failure, but it could also optimize postoperative recovery and functional and clinical outcomes [17].

VSP uses cross-sectional images of the affected area and CAD software to plan, simulate and validate the surgical procedure [30]. The main advantage of VSP is the ability to plan the surgical procedure in detail in a virtual environment, allowing accurate 3D visualization of the lesion and surrounding anatomical structures. This not only allows for the precise definition of resection margins and the design of implants that perfectly fit the individual patient’s anatomy but also helps the surgeon to implement these elements during the subsequent surgical phase, improving both the surgical outcome and the patient’s QOL [17,18,20,21,24,30]. The current literature shows a significant lack of prospective studies evaluating the efficacy of this innovative methodology in the treatment of canine CSA [31]. The use of CAD software reported in this study was simple and allowed the visualization of all bone planes (sagittal, frontal and transverse), assessment of the tumor and planning and simulation of the corrective ostectomy by calculating the exact site and margin of bone resection. Re-evaluation of the bone after surgery allowed the comparison of preoperative, expected and postoperative results to assess the effectiveness of the surgery. The ability to quickly change the standardized views (sagittal, frontal and transverse) made it easy to understand the effect of the planned surgery. During the VSP and 3D design of the PSIs, the surgeon drew the shape of the PSGs on the bone model using a brush tool to indicate the specific anatomical landmarks to be considered in relation to the chosen surgical approach. On the femur, the landmark for osteotomy guide placement was planned laterally and proximally at the level of the third trochanter, in a one-way fit position. On the tibia, the landmarks were planned proximally on the medial part of the medial condyle and distally on the cranial edge of the tibia immediately distal to the tibial tuberosity, also in a one-way fit position. These easily recognizable landmarks allow the PSGs to adhere firmly and avoid extensive soft tissue dissection.

Three-dimensional-printed PSIs, such as PSGs and PSEs, are designed from CT scans based on the anatomical, geometric and biomechanical characteristics of the recipient. Unlike generic cutting guides, which are designed based on average reference values, PSGs allow for customized and more accurate bone cuts, reducing trauma to surrounding tissues, the use of intraoperative diagnostics and ultimately, the operative time [30]. In our study, the bioengineer designed the PSGs, and the surgeon approved the final design. For the PSE design, the surgeon selected the number, location and direction of screws in the proximal and distal bone segments. At this stage, the bioengineer’s suggestions on biomechanics, such as implant thickness, were particularly important in the design of the PSGs. Trial surgery on 3D-printed bone models to test both PSGs and PSE prior to surgery was not performed to avoid additional costs to the owners. In our study, the use of these guides resulted in more efficient surgery and reduced surgical time. The use of a titanium alloy PSE not only supports the structural integrity of the limb but also provides a more refined fit, which is essential for optimal healing. Titanium implants have been widely accepted in veterinary orthopedics due to their biocompatibility and strength, but also because they significantly reduce the risk of complications, such as implant failure or infection, which are critical factors in surgical success [24,32,33]. Loss of anatomical alignment during endoprosthetic fixation is one of the most serious and common complications following traditional limb-sparing surgery. In our study, the holes used to fix the PSGs during VSP were designed to coincide with the placement of the PSE anchoring screws, thus avoiding the loss of anatomical alignment. The use of both PSGs and PSE can reduce limb-sparing surgery time by 25–50% and may reduce the risk of implant failure [17]. Consistent with the literature and based on the personal experience of the surgical team, the use of both PSGs and PSE, as described in this study, reduced operative time by approximately 30%. The postoperative recovery in our patient was remarkable, with weight-bearing 48 h after surgery demonstrating improved mobility and minimal discomfort. Early mobilization and effective pain management strategies are important in achieving favorable outcomes [30]. In this case, the combination of VSP and PSIs ensured rapid recovery. Follow-up radiographs were planned at 3, 6 and 12 months to assess implant integrity, degree of osseointegration and bone healing. However, due to the owner’s financial and psychological limitations, the planned follow-up radiographs were not performed. The sedation required for the follow-up radiographs due to the patient’s aggressive behavior was a concern for the owner, who, aware of the risk of late metastases but satisfied with his animal’s clinical recovery, refused further diagnostic imaging, also due to economic constraints. Although it was not possible to assess implant integrity, degree of endoprosthetic osseointegration and bone healing by radiographic follow-up, post-mortem radiography or necropsy, the results of both telephone follow-up and clinical examination at another veterinary facility confirmed the absence of behavioral or gait changes, pain on palpation, visible swelling or lesions around the endoprosthesis. It can, therefore, be assumed that no implant failure occurred until the patient’s death. In our study, survival was 23 months (approximately 690 days). Different mean survival times (MSTs) have been reported in patients with appendicular chondrosarcoma treated by amputation alone. Obradovich et al. [1] reported an MST of 163 days, Popovitch et al. [25] reported an MST of 540 days and Waltman et al. [2] reported a longer MST of 2618 days. Although our results appear to fall somewhere between those reported in the literature for dogs treated by amputation alone, it is important to underline that this is a single case, making it difficult to draw definitive conclusions or make direct comparisons. Furthermore, our report represents the first case of chondrosarcoma treated with limb-sparing surgery, making direct comparison impossible due to the lack of similar data in the available literature. The wide variability in survival may be related to differences in patient selection criteria, biological characteristics of the tumors and treatment strategies used.

The production costs associated with PSI printing make the procedure more expensive and increase the waiting time for surgery. Waiting times depend on the surgeon, the bioengineer and the company and correspond to the design, production and shipping phases of the final product. It usually takes three to four weeks from the CT scan to the arrival of the PSIs [30]. In our study, the expected waiting time for the arrival of the PSIs, and thus for the performance of corrective surgery, was 28 days from VSP. Although chemotherapy does not show a significant increase in median survival, adjuvant chemotherapy was given, considering the 28-day waiting period between the CT scan and surgery, to minimize the risk of potential tumor growth and metastasis. However, the increase in surgical precision, the reduction in operating time and, therefore, the cost of anesthesia, but also the reduction in functional recovery time, lead to an increase in animal welfare and owner satisfaction.

Despite promising initial results, further research is needed to assess the potential superiority of limb-sparing surgeries using 3D-printed PSIs over traditional techniques. Future controlled multicentric prospective studies with larger sample sizes and longer follow-up periods would be beneficial to assess the overall QOL of treated dogs.

## 4. Conclusions

In our study, the combination of VSP and 3D-printed PSGs and PSE for limb-sparing surgery to treat femoral CSA in an eight-year-old dog with concomitant DJD of the elbows and hips resulted in optimal outcomes. The successful application of these technologies obtained in this study highlights their potential in veterinary orthopedic and oncological surgery. The ability to customize both the surgical approach and the implants to the individual patient represents a significant advance in the field and offers hope for improved limb preservation and functional recovery in the treatment of appendicular bone tumors.

## Data Availability

The original contributions presented in this study are included in the article. Further inquiries can be directed to the corresponding author.

## Supplementary Materials

The following supporting information can be downloaded at: https://www.mdpi.com/article/10.3390/ani15050673/s1, Video S1: Three-month follow-up; grade II lameness persists with some circumduction of the limb.

## References

[1] Farese J.P., Kirpensteijn J., Kik M., Bacon N.J., Waltman S.S., Seguin B., Kent M., Liptak J., Straw R., Chang M.N. (2009). Biologic behavior and clinical outcome of 25 dogs with canine appendicular chondrosarcoma treated by amputation: A Veterinary Society of Surgical Oncology retrospective study. Vet. Surg..

[2] Waltman S.S., Seguin B., Cooper B.J., Kent M. (2007). Clinical outcome of nonnasal chondrosarcoma in dogs: Thirty-one cases (1986–2003). Vet. Surg..

[3] Thompson K.G., Pool R.R., Meuten D.J. (2018). Tumors of bones. Tumors in Domestic Animals.

[4] Sylvestre A.M., Brash M.L., Atilola M.A.O., Cockshutt J.R. (1992). A case series of 25 dogs with chondrosarcoma. Vet. Comp. Orthop. Traumatol..

[5] Thompson K.G., Dittmer K.E., Meuten D.J. (2017). Tumors of Bone. Tumors in Domestic Animals.

[6] Wendland T.M., Seguin B., Duerr F.M. (2019). Retrospective Multi-Center Analysis of Canine Socket Prostheses for Partial Limbs. Front. Vet. Sci..

[7] Wendland T.M., Seguin B., Duerr F.M. (2023). Prospective evaluation of canine partial limb amputation with socket prostheses. Vet. Med. Sci..

[8] Kirpensteijn J., van den Bos R., van den Brom W.E., Hazewinkel H.A. (2000). Ground reaction force analysis of large breed dogs when walking after the amputation of a limb. Vet. Rec..

[9] Shimada M., Nagashima T., Michishita M., Yazawa D., Hara Y. (2023). Case report: Limb-sparing surgery of tibial chondrosarcoma with frozen autologous bone graft using liquid nitrogen in a dog. Front. Vet. Sci..

[10] Boston S.E., Johnson S.A., Tobias K.M. (2017). Musculoskeletal neoplasia and limb-sparing surgery. Veterinary Surgery: Small Animal.

[11] Mitchell K.E., Boston S.E., Kung M., Dry S., Straw R.C., Ehrhart N.P., Ryan S.D. (2016). Outcomes of limb-sparing surgery using two generations of metal endoprosthesis in 45 dogs with distal radial osteosarcoma. Vet. Surg..

[12] Liptak J.M., Dernell W.S., Ehrhart N., Lafferty M.H., Monteith G.J., Withrow S.J. (2006). Cortical allograft and endoprosthesis for limb-sparing surgery in dogs with distal radial osteosarcoma: A prospective clinical comparison of two different limb-sparing techniques. Vet. Surg..

[13] Burton J.H., Powers B.E., Biller B.J. (2016). Clinical outcome in canine appendicular chondrosarcoma: A retrospective analysis of 35 cases. Vet. Comp. Oncol..

[14] Selmic L.E., Burton J.H., Thamm D.H., Withrow S.J., Lana S.E. (2014). Comparison of carboplatin and doxorubicin-based chemotherapy protocols in 470 dogs after amputation for treatment of appendicular osteosarcoma. J. Vet. Intern. Med..

[15] Wong K.C., Kumta S.M., Geel N.V., Demol J. (2015). One-step reconstruction with a 3D-printed, biomechanically evaluated custom implant after complex pelvic tumor resection. Comput. Aided Surg..

[16] Chen X., Xu L., Wang W., Li X., Sun Y., Politis C. (2016). Computer-aided design and manufacturing of surgical templates and their clinical applications: A review. Expert Rev. Med. Devices.

[17] Timercan A., Brailovski V., Petit Y., Lussier B., Séguin B. (2019). Personalized 3D-printed endoprostheses for limb sparing in dogs: Modeling and in vitro testing. Med. Eng. Phys..

[18] Miller M.A., Davis R.W. (2020). Three-dimensional planning in veterinary surgical oncology: A systematic review. Front. Vet. Sci..

[19] Palmisano M.P., Demetriou J.L., Benigni L. (2020). Comparison of traditional and 3D-printed surgical guides in veterinary limb-sparing procedures. Vet. Surg..

[20] Crosse K.R., Worth A.J. (2021). Current applications of 3D printing in small animal veterinary surgery. Vet. Surg..

[21] DeTora M.D., Boudrieau R.J. (2022). Applications of 3D printing in veterinary orthopedic surgery. Vet. Clin. N. Am. Small Anim. Pract..

[22] Smith R.L., Johnson M.E. (2023). Patient-specific implants in veterinary orthopedic oncology: A prospective study of 25 cases. Vet. Surg..

[23] Koch D., Fischer M.S. (2020). Visual Assessment and Gait Analysis. Diagnosing Canine Lameness.

[24] Memarian P., Pishavar E., Zanotti F., Trentini M., Camponogara F., Soliani E., Gargiulo P., Isola M., Zavan B. (2022). Active Materials for 3D Printing in Small Animals: Current Modalities and Future Directions for Orthopedic Applications. Int. J. Mol. Sci..

[25] Popovitch C.A., Weinstein M.J., Goldschmidt M.H., Shofer F.S. (1994). Chondrosarcoma: A retrospective study of 97 dogs (1987–1990). J. Am. Anim. Hosp. Assoc..

[26] Johnson K.A. (2014). Piermattei’s Atlas of Surgical Approaches to the Bones and Joints of the Dog and Cat.

[27] Kirpensteijn J., van den Bos R., Endenburg N. (1999). Adaptation of dogs to the amputation of a limb and their owners’ satisfaction with the procedure. Vet. Rec..

[28] Séguin B., Pinard C., Lussier B., Williams D., Griffin L., Podell B., Mejia S., Timercan A., Petit Y., Brailovski V. (2020). Limb-sparing in dogs using patient-specific, three-dimensional-printed endoprosthesis for distal radial osteosarcoma: A pilot study. Vet. Comp. Oncol..

[29] Dickerson V.M., Coleman K.D., Ogawa M., Saba C.F., Cornell K.K., Radlinsky M.G., Schmiedt C.W. (2015). Outcomes of dogs undergoing limb amputation, owner satisfaction with limb amputation procedures, and owner perceptions regarding postsurgical adaptation: 64 cases (2005–2012). J. Am. Vet. Med. Assoc..

[30] Panichi E., Cappellari F., Burkhan E., Principato G., Currenti M., Tabbì M., Macrì F. (2024). Patient-Specific 3D-Printed Osteotomy Guides and Titanium Plates for Distal Femoral Deformities in Dogs with Lateral Patellar Luxation. Animals.

[31] Roberts S.M., Mitchell K.E. (2022). Current concepts in the surgical management of canine appendicular chondrosarcoma. J. Am. Vet. Med. Assoc..

[32] Szczęsny G., Kopec M., Politis D.J., Kowalewski Z.L., Łazarski A., Szolc T. (2022). A Review on Biomaterials for Orthopaedic Surgery and Traumatology: From Past to Present. Materials.

[33] Ziąbka M., Matysiak K., Cholewa-Kowalska K., Kyzioł A., Królicka A., Sapierzyński R., Januchta-Kurmin M., Bissenik I. (2023). In Vitro and In Vivo Studies of Antibacterial Coatings on Titanium Alloy Implants for Veterinary Application. Int. J. Mol. Sci..

